# Supplementary material for: The long non-coding RNA LUCAT1 is a negative feedback regulator of interferon responses in humans
Source: Nat Commun. 2020 Dec 11;11:6348. doi: 10.1038/s41467-020-20165-5 (PMC7733444; doi:10.1038/s41467-020-20165-5)
Supplement: Supplementary file 1 — Supplementary Information [file 41467_2020_20165_MOESM1_ESM.pdf]

Supplementary Information

for

The long non-coding RNA LUCAT1 is a negative feedback regulator of Interferon Responses in humans

Agarwal et al.

File contains:

Supplementary Figures and Supplementary Table

Supplementary Figure 1.

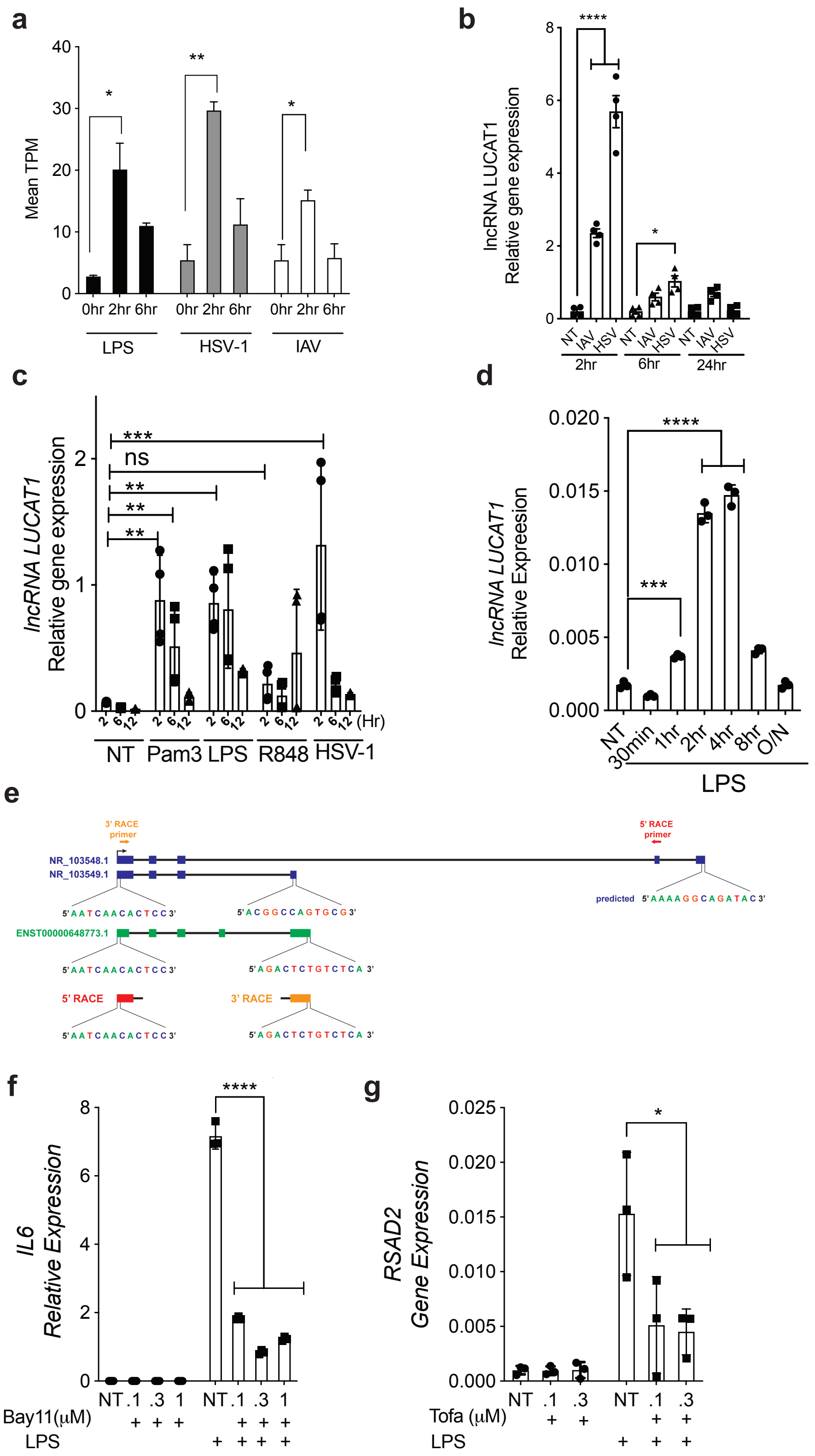

LUCAT1 is an inducible lncRNA upregulated in activated primary human cells upon immune stimulation
a LUCAT1 expression from RNA sequencing represented in TPM values for LPS, HSV-1 and IAV stimulated hDCs at 2hr and 6hr time points (n=2; biologically independent human sample, one-way ANOVA Dunnett's multiple comparisons test) **b** RT-qPCR analysis of LUCAT1 expression in hDCs stimulated with IAV and HSV-1 for 2hr, 6hr and 24hr time points (n=4; biologically independent human sample, one-way ANOVA Dunnett's multiple comparisons test, *0.0136; ****<0.0001). **c** RT-qPCR analysis of LUCAT1 expression in THP-1 cells stimulated with Pam3, LPS, R848 and HSV-1 for 2hr, 6hr and 12hr time points (n=4; biologically independent experiments, one-way ANOVA Dunnett's multiple comparisons test, LPS 2hr** 0.0027; LPS 6hr** 0.0045; Pam3 2hr** 0.0011;HSV-1 2hr*** 0.0006;R848=ns). **d** RT-qPCR analysis of LUCAT1 expression in BlaER1 cells stimulated with LPS from 30min to O/N time points (n=3; biologically independent experiments, one-way ANOVA Dunnett's multiple comparisons test; LPS 1hr *** 0.0002; LPS 2hr-4hr **** <0.0001). **e** Schematic showing sequences of identified 3' and 5' ends of LUCAT1 isoforms with RACE **f** RT-qPCR analysis of IL6 expression in primary hDC cells stimulated with 200ng/ml LPS and NFkB inhibitor Bay11 at .1uM, .3uM and 1uM concentrations (n=3; biologically independent human sample, one-way ANOVA Dunnett's multiple comparisons test; Bay11 .1uM, .3uM and 1uM **** <0.0001). **g** RT-QPCR analysis of Viperin expression in primary hDC cells stimulated with 200ng/ml LPS and JAK1 inhibitor Tofacitinib at .1 uM and .3 uM concentrations (n=3; biologically independent human sample, one-way ANOVA Dunnett's multiple comparisons test; Tofa .1uM * 0.0481 Tofa .3uM * 0.0385). Data is represented as mean ± SEM
* P ≤ 0.05, ** P ≤ 0.01, *** P ≤ 0.001,**** P ≤ 0.0001

Supplementary Figure 2.

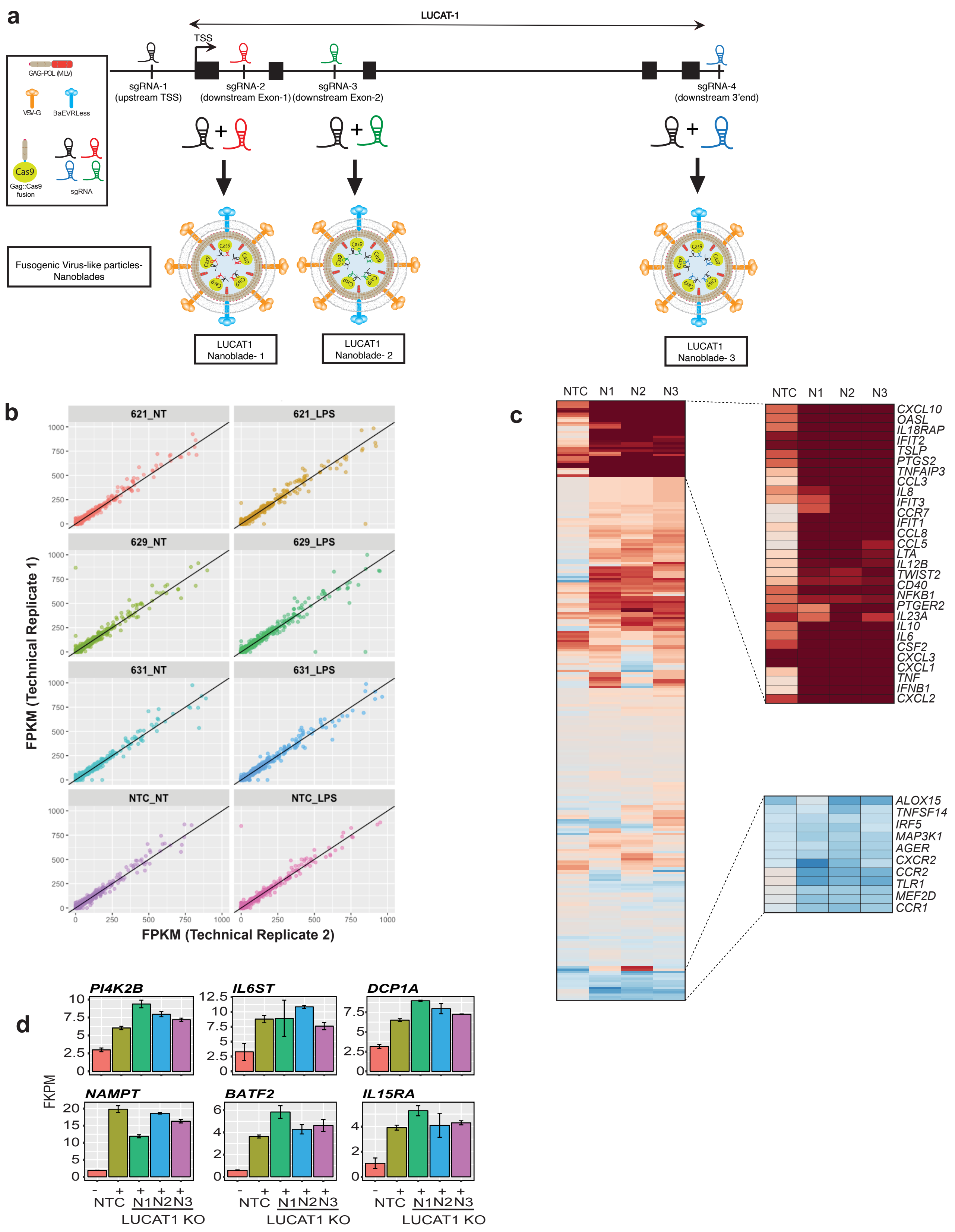

LUCAT1 deficiency leads to hyperactivation of an inflammatory and ISG signature

a Schematic showing LUCAT1 sgRNA encapsulated in Nanoblade VLPs. Three different combination of sgRNA were used to excise out LUCAT1. **b** Scatter plot analysis showing correlation between two RNA sequencing runs in Nanoblade mediated LUCAT1 targeted hDCs in NT and LPS stimulated conditions. **c** Heatmap analysis of inflammatory Nanostring code-set data representing the most differentially regulated genes in Nanoblade LUCAT1 hDCs compared to control in LPS stimulated conditions. **d** Bar graph representation of genes (in FPKM) that remain unchanged between Nanoblade targeted LUCAT1 hDCs and NTC controls in LPS stimulated conditions. Data is represented as mean ± SD.

Supplementary Figure 3.

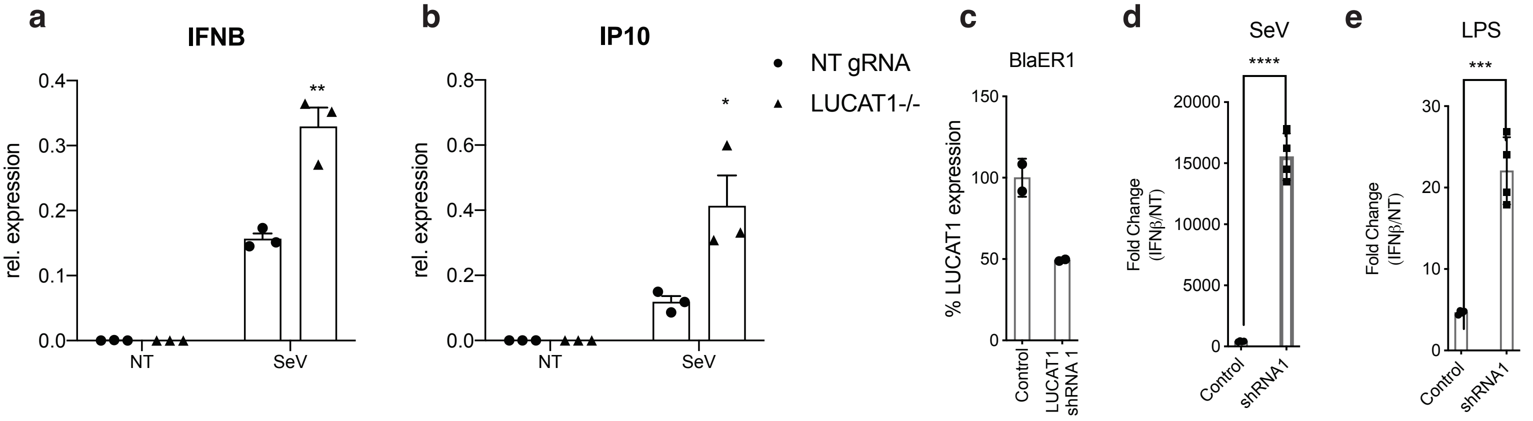

LUCAT1 deficiency leads to hyperactivation of an inflammatory and ISG signature

a, b RT-qPCR analysis of IP10 and IFNB expression in NT gRNA and LUCAT1-/- THP1 6 h after SeV infection (n=3; biologically independent experiments, unpaired two-tailed t-test; IP10: p=0.0363, IFNB: p=0.0049). **c** RT-qPCR analysis of LUCAT1 gene expression in LUCAT1 shRNA expressing BlaER1 cells upon LPS stimulation (n=2, biologically independent experiments). **d** RT-qPCR analysis of IFNβ expression represented as fold change over NT in LUCAT1 shRNA expressing BlaER1 cells upon SeV stimulation (n=4, biologically independent experiments, unpaired two-tailed t-test, p!<0.0001). **e** RT-qPCR analysis of IFNβ expression represented as fold change over NT in LUCAT1 shRNA expressing BlaER1 cells upon LPS stimulation (n=4; biologically independent experiments, unpaired two-tailed t-test, p= 0.0002). Data is represented as mean ± SEM * P ≤ 0.05, ** P ≤ 0.01, *** P ≤ 0.001,**** P ≤ 0.0001!

!

Supplementary figure 4.

A Primary human DC

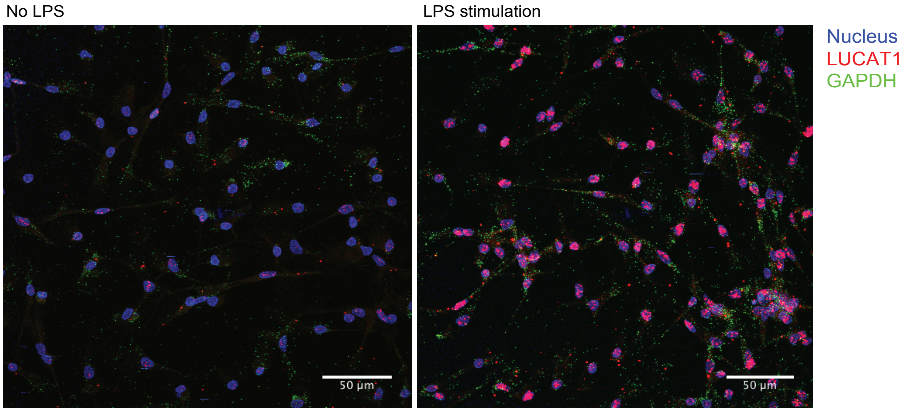

LncRNA LUCAT1 is enriched in nuclear compartment upon activation
a smFISH analysis for LUCAT1 in primary human hDCs in stimulated conditions. LUCAT1 probes are represented in red, chromatin staining by DAPI in blue and GAPDH mRNA in green (Scale 50μM). n=3, biologically independent experiments

Supplementary Table 1: RT-qPCR primer sequences

RT-qPCR primers	
huLUCAT1-F exon1	GCTCGGATTGCCTTAGACAG
huLUCAT1-R exon1	AAGAGTTCCAGCTGGGTGAG
huLUCAT1-F exon5	GCTCGGATTGCCTTAGACAG
huLUCAT1-R exon5	GGGTGAGCTTCTTGTGAGGA
huGap-F	TGCACCACCAACTGCTTA
huGap-R	AGAGGCAGGGATGATGTTC
huHPRT-F	ATCAGACTGAAGAGCTATTGTAATGA
huHPRT-R	TGGCTTATATCCAACACTTCGTG
huIFNB-F	GTCTCCTCCAAATTGCTCTC
huIFNB-R	ACAGGAGCTTCTGACACTGA
huIL6-F	TCTCCACAAGCGCCTTCG
huIL6-R	CTCAGGGCTGAGATGCCG
huTNF-F	CCTCTCTCTAATCAGCCCTCT
huTNF-R	GAGGACCTGGGAGTAGATGAG
HuIP10-F	GTGGCATTCAAGGAGTACCTC
HuIP10-R	TGATGGCCTTCGATTCTGGATT

Supplementary Table 2: Sequences of shRNA hairpin sequences for LUCAT1

shRNA hairpin sequences	
shRNA-Lucat1-E1-F	CCGGCCTTAGACAGGTGCAATTTAACTCGAGTTAAATTGCACCTGTCTAAGGTTTTTG
shRNA-Lucat1-E1-R	AATTCAAAAACCTTAGACAGGTGCAATTTAACTCGAGTTAAATTGCACCTGTCTAAGG
shRNA-Lucat1-E5-F	CCGGACACATTTCAGTCACTAAATACTCGAGTATTTAGTGACTGAAATGTGTTTTTTG
shRNA-Lucat1-E5-R	AATTCAAAAAACACATTTCAGTCACTAAATACTCGAGTATTTAGTGACTGAAATGTGT

Supplementary Table 3: LUCAT1 sgRNA sequences

Nanoblade sgRNA sequences	
ER-621 S	CACCGGTACATCGTTAGATTTGAAA
ER-622 AS	AAACTTTCAAATCTAACGATGTACC
ER-623 S	CACCGATATTGAAGCGAGCACCTCA
ER-624 AS	AAACTGAGGTGCTCGCTTCAATATC
ER-625 S	CACCGGACTTGCCACCCTGGTTGAT
ER-626 AS	AAACATCAACCAGGGTGGCAAGTCC
ER-627 S	CACCGGACGGCTGAAAATTGCTGAC
ER-628 AS	AAACGTCAGCAATTTTCAGCCGTCC
ER-629 S	CACCGAGGCTTCAAAGGGTTATGGG
ER-630 AS	AAACCCCATAACCCTTTGAAGCCTC
ER-631 S	CACCGGGGACGTGGGAGGCTTCAAA
ER-632 AS	AAACTTTGAAGCCTCCCACGTCCCC
ER-633 S	CACCGCATAGGTGACACCCACAAAT
ER-634 AS	AAACATTTGTGGGTGTCACCTATGC
CRISPRa sgRNA sequences	
LUCAT1-CRISPRa-L1-F	CACCGGTGACAAATCACATTGCCCT
LUCAT1-CRISPRa-L1-R	AAACAGGGCAATGTGATTTGTCACC
LUCAT1-CRISPRa-L2-F	CACCGAGAGATTGCCACAGACACCC
LUCAT1-CRISPRa-L2-R	AAACGGGTGTCTGTGGCAATCTCTC
LUCAT1-CRISPRa-L3-F	CACCGCACATTGCCCTGGGTGTCTG
LUCAT1-CRISPRa-L3-R	AAACCAGACACCCAGGGCAATGTGC
LUCAT1-CRISPRa-L4-F	CACCGGAGATTGCCACAGACACCCA
LUCAT1-CRISPRa-L4-R	AAACTGGGTGTCTGTGGCAATCTCC
LUCAT1-CRISPRa-L5-F	CACCGGATTTGTCACTTAAAGAGGA
LUCAT1-CRISPRa-L5-R	AAACTCCTCTTTAAGTGACAAATCC

CRISPR-cas9 sgRNA sequences used to generate THP-1 KO cells	
LUCAT1 sgFWD3	CACCGAGATTGCCACAGACACCCA
LUCAT1 sgREV3	AAACTGGGTGTCTGTGGCAATCTC
LUCAT1 sgREV6	AAACTGGTAGATGCTGAACCAATTC
LUCAT1 sgFWD6	CACCGAATTGGTTCAGCATCTACCA

Supplementary Table 4: List of LUCAT1 ChIRP Probes

LUCAT1 ChIRP Probe 1	TGAGAGAAAAGAGGATGAAAGCTGTTCTTAAATTGCACCTGTCTAAGGCAATCCGAGCTTGACACATGGTTTCTGGAGGTCTGGGCATTG
LUCAT1 ChIRP Probe 2	TCTCTGGTGCCAAGGTCCCATAAGAGTTCCAGCTGGGTGAGCTTCTTGTGAGGAAAGGAGCCAGAAGTCAGAACACATAGTGTGACAATA
LUCAT1 ChIRP Probe 3	AACCAATTTTGTTAACGTGAGAGAAATACAAGAAAGCCAAGTCAGAAATACCATTGTTGCTGTTAGAAAACTTCAAAGAGGAATTTGTGG
LUCAT1 ChIRP Probe 4	GCAGTGAACCGAGATCGCGCCACTGCACTCCAGCCTGGGCGACAGAGCGAAACTCTGTAGCTCAGCATGTAGCCCATGGTAGATGCTG
LUCAT1 ChIRP Probe 5	GGATTCCTGGGTGTGGTGGCGGGCGCCTGTAGTCCCAGCTACTCAGGAGGCTGAGGCAGGAGAATGGCGTGAACCCGGGAGGTGGAGCTT
LUCAT1 ChIRP Probe 6	CTCATCCTTCCAAAGACGTCAGTCACATTCAGCCCCTTTAGCAGTTTCATCAACAGCATGTATAGCACATGTGATAGCAAACAGCAAGTT
LUCAT1 ChIRP Probe 7	CTTATCTTCTGACATCTTCTGATGGGTTTTGTTCCTTTTCATTGGGAGATGAGGACAGCATTTGGACACAACTGTACAGGCACGCTAAGT
LUCAT1 ChIRP Probe 8	CCTCGGGTTGCCTCTGTTTATCCATCTCTCTTTTTTTAAGAAGTAGAACACTGAGGGACAGCTGGTAAGTGTAGCATCAGGACAAAAATC
LUCAT1 ChIRP Probe 9	AGGCTCTTTATTTGTGAGGGGATGAGAATACTGGCATCCATTGTGTCTTATTTAGTGACTGAAATGTGTGACACTGAGCAAGGCCTTTAT
