# Supplementary material for: The long non-coding RNA LUCAT1 is a negative feedback regulator of interferon responses in humans
Source: Nat Commun. 2020 Dec 11;11:6348. doi: 10.1038/s41467-020-20165-5 (PMC7733444; doi:10.1038/s41467-020-20165-5)
Supplement: Supplementary file 3 — Descriptions of Additional Supplementary Files [file 41467_2020_20165_MOESM3_ESM.pdf]

Descriptions of Additional Supplementary Files

Supplementary Data 1

Description: iBAQ values of proteins pulled down with LUCAT1 probes in ChIRP-MSiBAQ values of proteins pulled down with LUCAT1 probes in ChIRP-MS
