# Supplementary material for: The long non-coding RNA LUCAT1 is a negative feedback regulator of interferon responses in humans
Source: Nat Commun. 2020 Dec 11;11:6348. doi: 10.1038/s41467-020-20165-5 (PMC7733444; doi:10.1038/s41467-020-20165-5)
Supplement: Supplementary file 5 — Reporting Summary [file 41467_2020_20165_MOESM5_ESM.pdf]

nature research

Corresponding author(s): Kate A. Fitzgerald Ph.D

Last updated by author(s): 10/28/2020

Reporting Summary

Nature Research wishes to improve the reproducibility of the work that we publish. This form provides structure for consistency and transparency in reporting. For further information on Nature Research policies, see our Editorial Policies and the Editorial Policy Checklist.

Statistics

For all statistical analyses, confirm that the following items are present in the figure legend, table legend, main text, or Methods section.

n/a	Confirmed	
☐	☒	The exact sample size (*n*) for each experimental group/condition, given as a discrete number and unit of measurement
☐	☒	A statement on whether measurements were taken from distinct samples or whether the same sample was measured repeatedly
☐	☒	The statistical test(s) used AND whether they are one- or two-sided<br>*Only common tests should be described solely by name; describe more complex techniques in the Methods section.*
☒	☐	A description of all covariates tested
☒	☐	A description of any assumptions or corrections, such as tests of normality and adjustment for multiple comparisons
☐	☒	A full description of the statistical parameters including central tendency (e.g. means) or other basic estimates (e.g. regression coefficient) AND variation (e.g. standard deviation) or associated estimates of uncertainty (e.g. confidence intervals)
☐	☒	For null hypothesis testing, the test statistic (e.g. *F*, *t*, *r*) with confidence intervals, effect sizes, degrees of freedom and *P* value noted<br>*Give P values as exact values whenever suitable.*
☒	☐	For Bayesian analysis, information on the choice of priors and Markov chain Monte Carlo settings
☒	☐	For hierarchical and complex designs, identification of the appropriate level for tests and full reporting of outcomes
☒	☐	Estimates of effect sizes (e.g. Cohen's *d*, Pearson's *r*), indicating how they were calculated

Our web collection on statistics for biologists contains articles on many of the points above.

Software and code

Policy information about availability of computer code

Data collection	No custom software was used.
Data analysis	Paired-end sequence reads were aligned to the masked human genome using Bowtie and expression analysis was performed with RSEM and EBSeq . The RSEM-calculate expression program was run with --paired-end --forward-probe options and GTF version 84 from Ensembl. The EBSeq HMMTest function was used to calculate posterior probabilities for potential expression patterns in the time course experiment. The false discovery rate of genes was controlled at 5%, which corresponds to a posterior probability of 0.95 or greater. A pseudo value of one was added to TPM values prior to log transformations and calculation of fold-change values.<br>For RNA-seq on Nanoblades:<br>The sequence reads were aligned to human reference genome build hg19 using TopHat2 and Bowtie2 . FPKM (fragments per kilobase million) values were computed using Cufflinks and fold changes were calculated using Cuffdiff version 2.2.1. Gene Ontology enrichment was performed using DAVID [58]. Data analysis was implemented in R verion 3.6.0 statistical environment (http://www.r-project.org/).

For manuscripts utilizing custom algorithms or software that are central to the research but not yet described in published literature, software must be made available to editors and reviewers. We strongly encourage code deposition in a community repository (e.g. GitHub). See the Nature Research guidelines for submitting code & software for further information.

Data

Policy information about availability of data

All manuscripts must include a data availability statement. This statement should provide the following information, where applicable:

- Accession codes, unique identifiers, or web links for publicly available datasets
- A list of figures that have associated raw data
- A description of any restrictions on data availability

All accession codes for RNA sequencing data set submission are provided in the methods section. All the raw data supporting Fig 1(c, e, h), Figure 2(a, e), Fig 3 (a-h, j-l), Fig 4(a,c-d) Fig 5(a-i) and supplementary figures 1(a-f), supplementary figure 3(a-e) are provided in the Source Data file.

Field-specific reporting

Please select the one below that is the best fit for your research. If you are not sure, read the appropriate sections before making your selection.

☒ Life sciences ☐ Behavioural & social sciences ☐ Ecological, evolutionary & environmental sciences

For a reference copy of the document with all sections, see nature.com/documents/nr-reporting-summary-flat.pdf

Life sciences study design

All studies must disclose on these points even when the disclosure is negative.

Sample size	Sample size was chosen based on previous studies performed in the laboratory and in the lncRNA field.
Data exclusions	No data excluded
Replication	Data presented in the manuscript was reproducible in at least N=3 biological replicates.
Randomization	Cells were randomly assigned to experimental groups. They were also grouped based on genotype and treatment conditions. No additional randomization was applicable to the study.
Blinding	RNA sequencing was performed in a blinded fashion.

Reporting for specific materials, systems and methods

We require information from authors about some types of materials, experimental systems and methods used in many studies. Here, indicate whether each material, system or method listed is relevant to your study. If you are not sure if a list item applies to your research, read the appropriate section before selecting a response.

Materials & experimental systems

n/a	Involved in the study	
☐	☒	Antibodies
☐	☒	Eukaryotic cell lines
☒	☐	Palaeontology and archaeology
☒	☐	Animals and other organisms
☒	☐	Human research participants
☒	☐	Clinical data
☒	☐	Dual use research of concern

Methods

n/a	Involved in the study	
☒	☐	ChIP-seq
☒	☐	Flow cytometry
☒	☐	MRI-based neuroimaging

Antibodies

Antibodies used	RNA Polymerase II (RNA Pol-II; Active Motif #39097, used at 10ul per IP), Histone H3 trimethylated at Lysine 4 (H3K4me3; Abcam # ab8580, used at 2ug per 25ug of chromatin), STAT1 (Cell Signaling Technology, #9172, used at 1:50 dilution ratio), control IgG isotype (Abcam # ab37415 or Cell Signaling Technology #5415 at 1mg/ml concentration)
Validation	All the antibodies and each lot used have been validated by the manufacturer for the application of ChIP.<br>RNA Polymerase II (RNA Pol-II; Active Motif #39097)-https://www.activemotif.com/documents/tds/39097.pdf<br>Histone H3 trimethylated at Lysine 4 (H3K4me3; Abcam # ab8580)-https://www.abcam.com/histone-h3-tri-methyl-k4-antibody-chip-grade-ab8580.html<br>STAT1 (Cell Signaling Technology, #9172) https://www.cellsignal.com/products/primary-antibodies/stat1-antibody/9172?Ntk=Products&Ntt=9172<br>control IgG isotype-https://www.cellsignal.com/products/primary-antibodies/mouse-g3a1-mab-igg1-isotype-control/5415?Ntk=Products&Ntt=5415

Eukaryotic cell lines

Policy information about cell lines

Cell line source(s)	ATCC, THP-1 , HEK293T,<br>BlaER1 cells were obtained from Veit Hornung, Munich. The commercial source for BlaER1 is https://www.emdmillipore.com/US/en/product/BLaER1-Human-B-cell-Precursor-Leukemia-Cell-Line,MM_NF-SCC165
Authentication	Cell lines obtained from external vendors and institutions were authenticated by morphology, phenotype and growth.
Mycoplasma contamination	All cell lines were tested negative for mycoplasma contamination using Mycoplasma detection kit.
Commonly misidentified lines<br>(See ICLAC register)	No commonly misidentified cell lines were used.
